# Mitochondrial Dysfunctions May Be One of the Major Causative Factors Underlying Detrimental Effects of Benzalkonium Chloride

**DOI:** 10.1155/2020/8956504

**Published:** 2020-02-10

**Authors:** Anton G. Rogov, Tatyana N. Goleva, Evgeniya I. Sukhanova, Khoren K. Epremyan, Tatiana A. Trendeleva, Alexandra P. Ovchenkova, Dinara A. Aliverdieva, Renata A. Zvyagilskaya

**Affiliations:** ^1^Federal Research Center “Fundamentals of Biotechnology”, Russian Academy of Sciences, Leninsky pr. 33, 119071 Moscow, Russia; ^2^Caspian Institute of Biological Resources of the Russian Academy of Sciences, M. Gadzhiev st. 45, Makhachkala, 367025, Russia

## Abstract

Benzalkonium chloride (BAC) is currently the most commonly used antimicrobial preservative in ophthalmic solutions, nasal sprays, and cosmetics. However, a large number of clinical and experimental investigations showed that the topical administration of BAC-containing eye drops could cause a variety of ocular surface changes, from ocular discomfort to potential risk for future glaucoma surgery. BAC-containing albuterol may increase the risk of albuterol-related systemic adverse effects. BAC, commonly present in personal care products, in cosmetic products can induce irritation and dose-dependent changes in the cell morphology. The cationic nature of BAC (it is a quaternary ammonium) suggests that one of the major targets of BAC in the cell may be mitochondria, the only intracellular compartment charged negatively. However, the influence of BAC on mitochondria has not been clearly understood. Here, the effects of BAC on energy parameters of rat liver mitochondria as well as on yeast cells were examined. BAC, being a “weaker” uncoupler, potently inhibited respiration in state 3, diminished the mitochondrial membrane potential, caused opening of the Ca2+/Pi-dependent pore, blocked ATP synthesis, and promoted H_2_O_2_ production by mitochondria. BAC triggered oxidative stress and mitochondrial fragmentation in yeast cells. BAC-induced oxidative stress in mitochondria and yeast cells was almost totally prevented by the mitochondria-targeted antioxidant SkQ1; the protective effect of SkQ1 on mitochondrial fragmentation was only partial. Collectively, these data showed that BAC acts adversely on cell bioenergetics (especially on ATP synthesis) and mitochondrial dynamics and that its prooxidant effect can be partially prevented by the mitochondria-targeted antioxidant SkQ1.

## 1. Introduction

**Figure 1:**
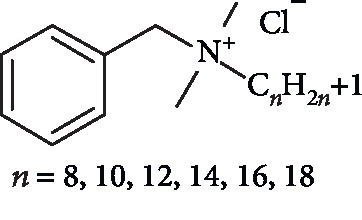
Structure of BAC.

Benzalkonium chloride (BAC) (its chemical structure is shown in Figure 1) is a synthetic quaternary ammonium compound with amphoteric surface-acting properties [1]. It is a mixture of several alkyldimethylbenzylammonium chlorides differing only in the length of their alkyl side chains; commercially available BAC consists of benzyldodecyldimethylammonium chloride, benzyldimethyltetradecylammonium chloride, and benzylcetyldimethylammonium chloride [2]. BAC, a membrane-active cationic surfactant, is remarkable by its stability, high water solubility, low cost, and superior biocidal activity against most bacteria, fungi, algae, and some viruses [3–6]. It is extensively employed as an antimicrobial in hospitals, agriculture, and the food industry [4], in ophthalmic preparations [7], nasal sprays [8], multidose albuterol nebulizer solutions [9, 10], as a skin disinfectant and hand sanitizer [11], in cosmetics, numerous toothpastes and mouth rinses, lozenges, and dental restoratives [12–16].

The mechanism of antimicrobial action of BAC is still unknown in detail, but there is concern that biocide effect may occur through ionic and hydrophobic interactions, the increased expression of efflux pumps with the subsequent leakage of intracellular constituents, ultimately leading to cell death [2, 17]. The BAC-mediated surplus of ROS might lead to DNA damage and a decrease in glutathione, suggesting an apoptotic phenomenon [18]. The mechanism of action of BAC on fungal cells, including yeasts, is believed to be similar to the antibacterial activity [17]. It should, however, be recognized that only limited work has been dedicated to assess this issue, which remains not clearly understood.

Although BAC provides excellent antimicrobial properties in ophthalmic preparations, a large number of clinical and experimental investigations using *in vitro* or animal models suggested cytotoxic effects of even low BAC concentrations on several components of the eye (see [18]). The topical administration of BAC-containing eye drops may cause a variety of ocular surface changes, from ocular discomfort, redness, dryness, and tear film instability [19–26] to allergic, immune, inflammatory reactions [27–29], ocular irritation, scarring of the ocular surface with irreversible vision impairment [21, 30, 31], disruption of the blood-aqueous barrier inducing cystoid macular edema following cataract surgery [32, 33], loss of goblet cells (see [27, 34]) and, at higher concentrations, to the disruption of the corneal epithelium, induction of apoptosis or necrosis of Chang's conjunctival cells [31–33, 35, 36]. The proapoptotic effects were seen at very low concentrations of BAC with a threshold of toxicity found at about 0.005% (i.e., below the usual concentration used in most eye drops). In addition, ROS production in preserved formulations was at significantly higher levels than those observed with unpreserved drugs [31].

BAC added to the multidose albuterol nebulizer solution as an antimicrobial produced bronchospasm that was dose-dependent and cumulative [37]. Moreover, the use of BAC-containing albuterol during severe acute asthma exacerbations may antagonize the bronchodilator response to albuterol and increase the risk of albuterol-related systemic adverse effects [9].

Long-term use of intranasal corticosteroids with BAC can lead to high-grade dysplasia in the nasal mucosa [38].

BAC, commonly present as a preservative in personal care and cosmetic products, can induce irritation, predisposal to sensitization in dermatitis patients [39–41], dose-dependent changes in the cell morphology, and at higher concentrations neural toxicity through ROS induction and apoptosis [12], and even cell atrophy [13].

At the cellular level, in neuroblastoma cells [42] and the mouse neonatal brain [43], BAC exposure significantly modified lipid homeostasis changing several lipid classes. Long-term exposure of *Escherichia coli* to environmentally relevant BAC concentrations resulted in significant alterations in global gene expression with increasing the expression of genes associated with efflux and reducing the expression of genes associated with outer-membrane porins, motility, and chemotaxis [44]. In cultured human alveolar epithelial cells, BAC induced a dose- and concentration-dependent oxidative stress [45]. At high concentrations, BAC brought about neural toxicity in rat brains through ROS induction and apoptosis [12].

However, the cationic nature of BAC (it is a quaternary ammonium) suggests that one of the major targets of BAC in the cell may be mitochondria, the only intracellular compartment charged negatively. Indeed, a short-term exposure of Chang's conjunctival cells to BAC at concentrations ranging between 0.0001% and 0.01% significantly decreased the fluorescent intensity of Rhodamine 123, a membrane potential-related fluorescent probe, suggesting partial mitochondrial dysfunction, supposedly due to the opening of mitochondrial permeability transition pores; simultaneously, an enhanced hydrogen peroxide production was observed under these conditions [32]. In mice having a mutated mitochondrial complex II (succinate dehydrogenase) of the respiratory chain with overproduction of superoxide anion radicals, mitochondria-induced oxidative stress can be a causative factor for the development of dry eye disease [46]. Moreover, mitochondrial oxidative stress can influence pathogenesis and progression of age-related corneal diseases, as well as generalized corneal aging [47]. BAC at pharmacologically relevant concentrations inhibited mitochondrial ATP production and oxygen consumption in human corneal epithelial cells and cells bearing LHON mutations and osteosarcoma cybrid cells, possibly by directly targeting mitochondrial complex I [48, 49]. The authors concluded that the findings obtained support the urgent need for additional research on the mitochondria-related inhibitory effects of BAC [49]. Since mitochondria play a pivotal role in cellular functions, exact identification of molecular targets of BAC in mitochondria may greatly assist in a better understanding of biochemical mechanisms underlying its adverse effects.

In this study, we comprehensively analyzed BAC effects on energy parameters of isolated tightly coupled rat liver mitochondria, which are a perfect model for such kind of these investigations as they combine relative simplicity of their isolation with obtaining sufficient amounts of high-quality organelles. Other model organisms used in this study were yeasts *Yarrowia lipolytica* and *Dipodascus magnusii* with the respiratory chains closely resembling those of higher organisms [50, 51]. The *Y. lipolytica* yeast, an obligate aerobe amenable to both classical and molecular genetic techniques, and the *D. magnusii* giant cells containing branched mitochondrial network (see [52]) are exceptionally useful models for deciphering effects exerted by biologically active compounds on cell metabolism and mitochondrial dynamics. The benefits of humanized yeast models to provide insight into the mechanisms of key biochemical processes have been repeatedly documented (see [53–57]).

BAC was found to have deleterious effects on mitochondrial functioning causing collapse of the membrane potential, promoting membrane permeabilization, inhibiting ATP synthesis, and promoting hydrogen peroxide production. In yeast cells, BAC triggered oxidative stress and mitochondrial fragmentation; its prooxidant effect can be partially prevented by the mitochondria-targeted antioxidant.

## 2. Materials and Methods

### 2.1. Chemical Reagents

Adenylate kinase, aminotriazole, Amplex Red, Ap5A, ATP, carbonylcyanide m-chlorophenylhydrazone (CCCP), cyclosporine A (CsA), EGTA, fatty acid-free BSA, glutamate, glucose-6-phosphate dehydrogenase, glycerol, hexokinase, malate, succinate, sucrose, mannitol, rotenone, NADP, phosphoenolpyruvate, pyruvate kinase, MgCl_2_, (NH_4_)_2_SO_4_, NaCl, Phenol red, and Tris were purchased from Sigma-Aldrich (USA). BAC (as a mixture of BACs with an average mol. weight of 360 and benzyldimethyldodecylammonium chloride as the prevalent species present) was from Fluka (Germany); Coomassie G-250 was from MP Biomedicals (USA); safranin O, KH_2_PO_4_, K_2_HPO_4_, KCl, and CaCl_2_ were from Merck (Germany); MitoTracker Green FM, and H_2_DCF-DA, and propidium iodide were from Life Technologies (USA). Other reagents of the highest quality available were from domestic suppliers. SkQ1 was a gift from Dr. G. A Korshunova.

### 2.2. Isolation of Rat Liver Mitochondria

In this study, adult (200–250 g) Wistar albino rats were used. The animal handling and mitochondria isolation were as described in [58].

### 2.3. Oxygen Consumption

Oxygen consumption was monitored amperometrically at 25°C using a Clark-type oxygen electrode [58]. The basic incubation medium containing 0.21 M mannitol, 0.09 M sucrose, 2 mM Tris-phosphate, pH 7.2, mitochondria (0.5 mg/ml), and either 20 mM Tris-succinate+rotenone (2 *μ*g/mg protein) or 20 mM Tris-glutamate, 5 mM Tris-malate was supplemented with 0.5 mM EGTA. Respiratory control ratios were calculated according to [59].

### 2.4. Transmembrane Potential Generation (Δ*ψ*)

Potential generation on the inner mitochondrial membrane (Δ*ψ*) was recorded as described in [58].

### 2.5. Swelling of Mitochondria

Swelling of mitochondria was recorded with a Varian Cary 300 Bio spectrophotometer (USA) by tracing the decrease in apparent absorbance of the mitochondrial suspensions at 540 nm [58].

### 2.6. Opening of the Nonspecific Ca^2+^/Pi-Dependent Pore

Opening of the nonspecific Ca^2+^/Pi-dependent pore (mPTP) was judged from the combination of two parameters [60], i.e., from the membrane potential depolarization and large-scale swelling of the mitochondria isolated and incubated in EGTA-free media.

### 2.7. ATP Synthesis by Mitochondria

ATP synthesis by rat liver mitochondria was monitored by two independent methods. The first one is based on the use of a pH-dependent dye Phenol red to follow small pH shifts during ADP conversion to ATP [61]. The basic incubation medium was supplemented with 0.5 mM EGTA, 6 *μ*M Ap5A (the inhibitor of adenylate kinase), 25 *μ*M Phenol red, and mitochondria (0.5 mg mitochondrial protein/ml). All stock solutions and additives were carefully adjusted to pH 7.1. Synthesis of ATP was induced by the addition of 500 *μ*M ADP and assayed spectrophotometrically at 557/618 nm with a Beckman Coulter DU-650 spectrophotometer (USA).

The second method, being complementary to the first one, is based on the association of ATP synthesis with NADP reduction in enzymatic reactions involving NADP, hexokinase, and glucose-6-phosphate dehydrogenase. The basic incubation medium was supplemented with 0.5 mM EGTA, 6 *μ*M Ap5A, 1 mM glucose, 1 mM NADP, hexokinase (10 IU/ml), glucose-6-phosphate dehydrogenase (3 IU/ml), and mitochondria (0.5 mg protein/ml). The synthesis of ATP was initiated by adding 100 *μ*M ADP and monitored with a Varian Cary 300 Bio (USA) spectrophotometer at 340 nm.

### 2.8. Hydrogen Peroxide Production by Mitochondria

Hydrogen peroxide production by mitochondria was determined fluorometrically by measuring oxidation of Amplex Red coupled to the enzymatic reduction of H_2_O_2_ by horseradish peroxidase (HRP) as described in [58]. The incubation medium was supplemented with 0.5 mM EGTA, 20 mM Tris-succinate, 6 mM aminotriazole (an inhibitor of catalase), 5 *μ*M Amplex Red, 9 U/ml horseradish peroxidase, and mitochondria (0.25 mg protein/ml). Where indicated, 1.5 *μ*M antimycin A (an inhibitor of electron transport in complex III of the respiratory chain) and 3 *μ*M SkQ1 were added. Fluorescence signal from a well containing all substrates and inhibitors, but not mitochondria, was subtracted as a background for every experimental condition used. Thus, any nonenzymatic effect of the inhibitors on apparent ROS production was eliminated.

### 2.9. Assay of Mitochondrial Protein

Mitochondrial protein was assayed by the Bradford method [62] with BSA as a standard.

### 2.10. Yeast Cell Cultures

In this study, we used the *Yarrowia lipolytica* strain obtained by RZ [63] and the *Dipodascus* (formerly *Endomyces*) *magnusii* yeast, strain VKM Y261. Cells were cultivated as described in [58].

### 2.11. ROS Determination in Yeast Cells

Production of intracellular reactive oxygen species (ROS) was detected using the nonfluorescent cell-permeating compound, 2′-7′-dichlorofluorescein diacetate (DCF-DA) as described in [58]. DCF-DA is hydrolyzed by intracellular esterases and then oxidized by ROS to a fluorescent compound, 2′-7′-dichlorofluorescein (DCF), detecting primarily hydrogen peroxide. Exponentially grown *Y. lipolytica* cells were either incubated with 10 *μ*M BAC for 2 h or initially preincubated with 3 *μ*M SkQ1 for 30 min and then incubated with 10 *μ*M BAC for 2 h. All samples were rinsed with a fresh portion of growth medium and incubated with 15 *μ*M H_2_DCF-DA for 30 min. Stained cells were analyzed for DCF detection by flow cytometry with a Becton & Dickinson FACSCalibur flow cytometer (USA).

### 2.12. Visualization of Mitochondria in Cells

Mitochondria were stained with the 200 nM fluorescent probe MitoTracker Green FM (Life Technologies, USA) that labels mitochondria in a membrane potential-independent manner. Stained cells were observed under the Olympus BX51 fluorescence microscope (Japan) equipped with 100x UPlanFL Oil objective (NA 1.30). Details are given in figure legends.

### 2.13. Time-Lapse Microscopy

For time-lapse microscopy (see [64]), *D. magnusii* cells harvested at the exponential growth phase were washed with 50 mM PBS, pH 5.5, and stained with 200 nM MitoTracker Green FM for 30 min. Stained cells were resuspended in a fresh portion of growth medium (the control variant), exposed to 45 *μ*M BAC, and observed under the Olympus BX51 microscope (Japan) equipped with 100x UPlanFL Oil objective (NA 1.30) for the times indicated. Images were acquired at specified intervals using Olympus DP30BW CCD camera (Japan). Images were analyzed with Icy software [65].

### 2.14. 3D Reconstruction of Mitochondrial Structures in *D. magnusii*

For 3D reconstruction, *D. magnusii* cells harvested at the exponential growth phase were incubated in a fresh portion of incubation medium (the control variant) or exposed to 45 mM BAC for 1 hour, then washed with 50 mM PBS, pH 5.5, and stained with 200 nM MitoTracker Green FM. Imaging was performed using an inverted motorized microscope Nikon Eclipse Ti-E with Perfect Focus autofocusing system (Japan). The microscopy system was equipped with 100x Apo TIRF Oil objective (NA1.49) and cooled EM-CCD camera Andor iXonDU-897E (United Kingdom) under the control of NIS-Elements 4.0 software. Serial optical sections were deconvolved using the AutoQuant blind deconvolution algorithm included in the NIS-Elements package. 3D reconstruction was performed by using Icy software [65].

### 2.15. Statistical Analysis

Unless otherwise specified, all experiments with isolated rat liver mitochondria were performed at least four times with consistent results. For analysis of mitochondrial morphology, at least fifty cells were examined in each trial. Data are presented as *X*_mean_ ± SD; *n* = 4. The statistical analyses were carried out by the one-way ANOVA test.

## 3. Results

### 3.1. Characteristics of Rat Liver Mitochondria Preparations

Preparations of rat liver mitochondria used in this study met all known criteria of physiological integrity as inferred from their ability to maintain distinctive state 4–3 respiration transitions upon successive additions of ADP, high respiratory rates, high respiratory control ratios ranging from 8 to 13 upon oxidation of NAD-dependent substrates, ADP/O ratios close to the theoretically expected maxima for the substrates studied, and some additional tests including an ability to maintain the substrate-supported membrane potential at the highest possible level for a long time; stimulation of respiration in state 4 by addition of AMP instead of ADP in the Mg^2+^-free incubation medium, which is indicative of preserving appreciable amounts of intramitochondrial Mg^2+^ and adenylate kinase largely located in the intermitochondrial space; cytochrome *c* test in evaluation of the intactness of the outer mitochondrial membrane.

### 3.2. Effect of BAC on Oxygen Consumption by Rat Liver Mitochondria in State 4 Respiration

**Figure 2:**
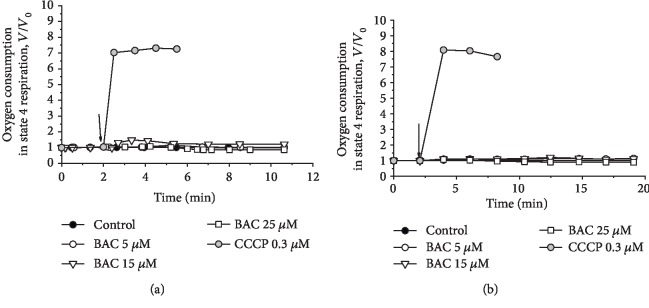
Effect of CCCP and varying BAC concentrations on state 4 respiration (*V*) of rat liver mitochondria respiring on (a) succinate or (b) glutamate+malate. First derivatives of amperometric curves are shown. The basic incubation medium was supplemented with 0.5 mM EGTA, mitochondria (0.5 mg protein/ml), and either (a) 20 mM Tris-succinate+rotenone (2 *μ*g/mg protein) or (b) 20 mM Tris-glutamate and 5 mM Tris-malate. Additions and amounts are given on the figures. Arrows point to the addition of CCCP and varying BAC concentrations. *V*_0_ is defined as respiratory rate in state 4 respiration before the addition of CCCP and varying BAC concentrations (24.8 or 11.2 ng-atom O/min per mg protein upon oxidation of succinate or glutamate+malate, respectively).

At moderate concentrations, BAC only slightly and transiently stimulated oxidation of succinate (Figure 2(a)) or NAD-dependent substrates (glutamate and malate) (Figure 2(b)) in state 4 respiration. The classical anionic uncoupler CCCP increased the rate of substrate oxidation in state 4 several fold, usually to an extent comparable to the respiratory control ratio. As the effect exerted by BAC was developed with time, we displayed in the figures the first derivatives of amperometric curves of oxygen consumption. Thus, BAC did not exhibit an uncoupling activity, resembling in this respect the action of other hydrophobic quaternary ammonium compounds such as decyltriethylammonium bromide (C_10_TEA) and cetyltrimethylammonium bromide (C_16_TMA) investigated previously [66].

### 3.3. Effect of BAC on Oxygen Consumption by Rat Liver Mitochondria in State 3 and Uncoupled Respiration

**Figure 3:**
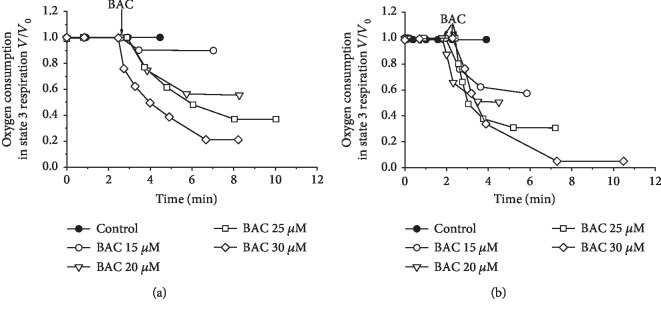
Inhibitory effect of varying BAC concentrations on state 3 respiration (*V*) of rat liver mitochondria respiring on (a) succinate or (b) glutamate+malate. First derivatives of amperometric curves are shown. The basic incubation medium was supplemented with 0.5 mM EGTA, mitochondria (0.5 mg protein/ml), and either (a) 20 mM Tris-succinate+rotenone (2 *μ*g/mg protein) or (b) 20 mM Tris-glutamate and 5 mM Tris-malate. State 3 respiration was initiated by the addition of 0.9 mM ADP. Additions and amounts are given on the figures. Arrows point to the additions of varying BAC concentrations. *V*_0_ is defined as respiratory rate in state 3 (in the presence of ADP) before the addition of varying BAC concentrations (172.4 or 89.8 ng-atom O/min per mg protein upon oxidation of succinate or glutamate+malate, respectively).

In the presence of ADP (in state 3 respiration), BAC inhibited oxidation of both succinate (Figure 3(a)) and NAD-dependent substrates (Figure 3(b)). In contrast, in the uncoupled state (in the presence of CCCP), BAC only slightly inhibited respiration supported by succinate or NAD-dependent substrates (not shown), suggesting that the main targets of BAC may be the translocase of adenine nucleotides or/and ATP synthase. As will be shown below, some of these assumptions proved to be justified.

### 3.4. BAC-Induced Depolarization of Rat Liver Mitochondria

**Figure 4:**
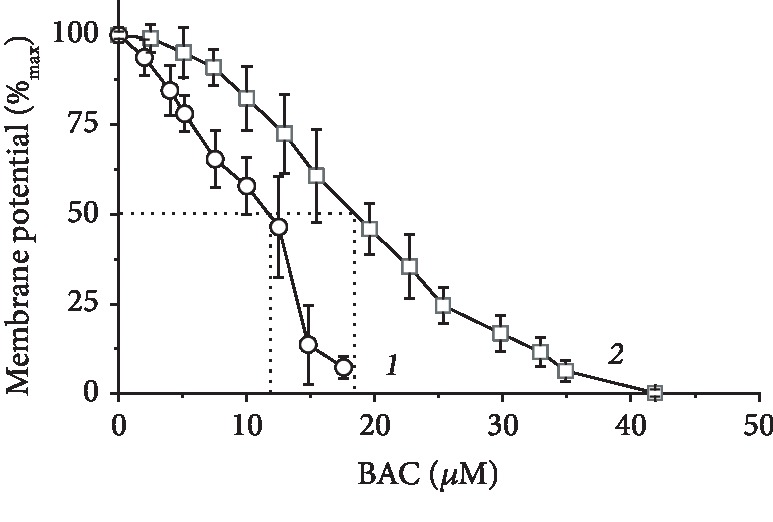
Membrane depolarization by BAC. The basic incubation medium was supplemented with 0.5 mM EGTA, 20 mM safranin O, mitochondria (0.5 mg protein/ml), and either 20 mM Tris-glutamate and 5 mM Tris-malate (curve *1*) or 20 mM Tris-succinate+rotenone (2 *μ*g/mg protein) (curve *2*).

BAC addition caused depolarization of mitochondria (Figure 4), the depolarizing effect being much stronger upon oxidation of NAD-dependent substrates (Figure 4(a), curve *1*) than in the case of succinate oxidation (Figure 4(a), curve *2*). The BAC-induced membrane depolarization was not reversed by the addition of ATP (not shown). In our previous work comprising direct trials [67], we argued that the ATP-induced recovery of the membrane potential after depolarization by a tested compound provides evidence against the membrane damage or disruption of ATP synthesis. The lack of ATP-related recoupling after the dissipation of membrane potential by BAC addition supported the view that BAC may exert a damaging effect on mitochondrial bioenergetics or even on mitochondrial membrane integrity.

### 3.5. BAC-Induced Opening of the mPTP in Rat Liver Mitochondria

**Figure 5:**
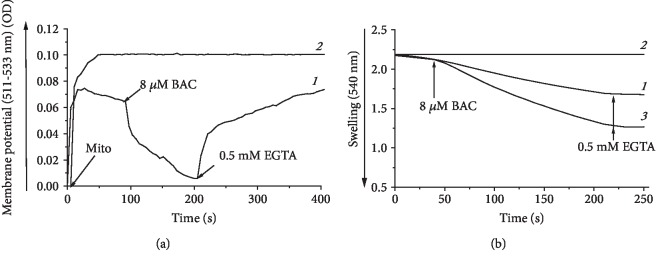
BAC promoted opening of the mPTP, as inferred from recording of the (a) membrane potential and (b) swelling of rat liver mitochondria isolated in EGTA-free medium. The basal incubation medium was supplemented with 20 mM Tris-succinate+rotenone (2 *μ*g/mg protein), mitochondria (0.5 mg protein/ml), and either (a) 20 mM safranin or (b) 40 mM KCl. (a, b) Curves *1*, spontaneous depolarization (a, curve *1*) and swelling (b, curve *1*) of rat liver mitochondria; (a, b) curves *2*, the media initially contained 0.5 mM EGTA; in (a), curve *1*, depolarizing effect of 8 *μ*M BAC and repolarization after the subsequent addition of 0.5 mM EGTA; in (b), curve *3*, promotion of mitochondrial swelling by 8 *μ*M BAC and subsequent compression of mitochondria after the addition of 0.5 mM EGTA at the end of incubation.

Rat liver mitochondria isolated and suspended in EGTA-free medium exhibited spontaneous decline in the membrane potential (Figure 5(a), curve *1*) and swelling (Figure 5(b), curve *1*) presumably because of the opening of the Ca^2+^/Pi-dependent, mildly anionic pore known as the mitochondrial permeability transition pore (mPTP) and mPTP-related unrestricted entry of osmotically active solutes into the mitochondrial matrix followed by the water uptake. These spontaneous membrane depolarization and swelling were completely inhibited in the presence of 0.5 mM EGTA or CsA (1.8 *μ*g/mg protein) (not shown) added at zero time (Figures 5(a) and 5(b), curve *2*), thus supporting the fact that the initial spontaneous partial opening of the mPTP took place. 8 *μ*M BAC caused collapse of the membrane potential (Figure 5(a), curve *1*) and increased the amplitude of swelling (Figure 5(b), curve *3*), which most likely reflected promotion of the mPTP opening.

### 3.6. BAC-Induced Inhibition of ATP Synthesis by Rat Liver Mitochondria

**Figure 6:**
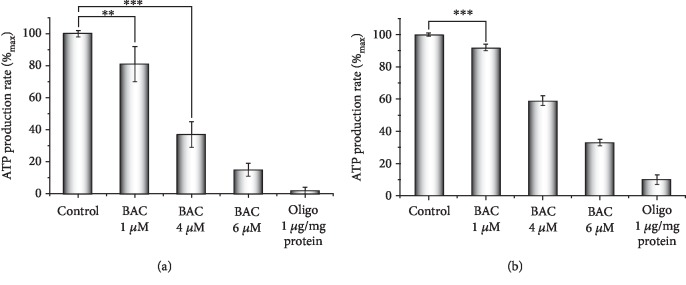
Inhibitory effect of BAC on ATP production by rat liver mitochondria respiring on succinate. The basal incubation medium was supplemented with 0.5 mM EGTA, 6 *μ*M Ap5A, an inhibitor of adenylate kinase, 20 mM Tris-succinate+rotenone (2 *μ*g/mg protein), and either (a) 25 *μ*M Phenol red and mitochondria (0.5 mg protein/ml) or (b) 1 mM glucose, 1 mM NADP, 10 U/ml hexokinase, 3 U/ml glucose-6-phosphate dehydrogenase, and mitochondria (0.25 mg protein/ml). Where indicated, oligomycin (Oligo, 1 *μ*g/mg protein) and varying BAC concentrations were added. The statistical analyses were carried out by the one-way ANOVA test. ^∗∗∗^*p* < 0.001; ^∗∗^0.001 < *p* < 0.01.

ATP synthesis was assayed in rat liver mitochondria respiring on succinate by two independent methods (for details, see Materials and Methods) with similar results (Figures 6(a) and 6(b)). To prevent mPTP opening, the basic incubation medium was supplemented with 0.5 mM EGTA; 6 *μ*M Ap5A, an inhibitor of adenylate kinase activity, was also added. Oligomycin, a well-known inhibitor of ATP synthesis in mitochondria, was used as a positive control. BAC was found to be a powerful inhibitor of ATP synthase sufficiently diminishing ATP synthesis even at very low concentrations not inhibiting state 3 respiration and only nominally depolarizing the membrane potential.

### 3.7. BAC-Induced Promotion of Hydrogen Peroxide Production by Rat Liver Mitochondria

**Figure 7:**
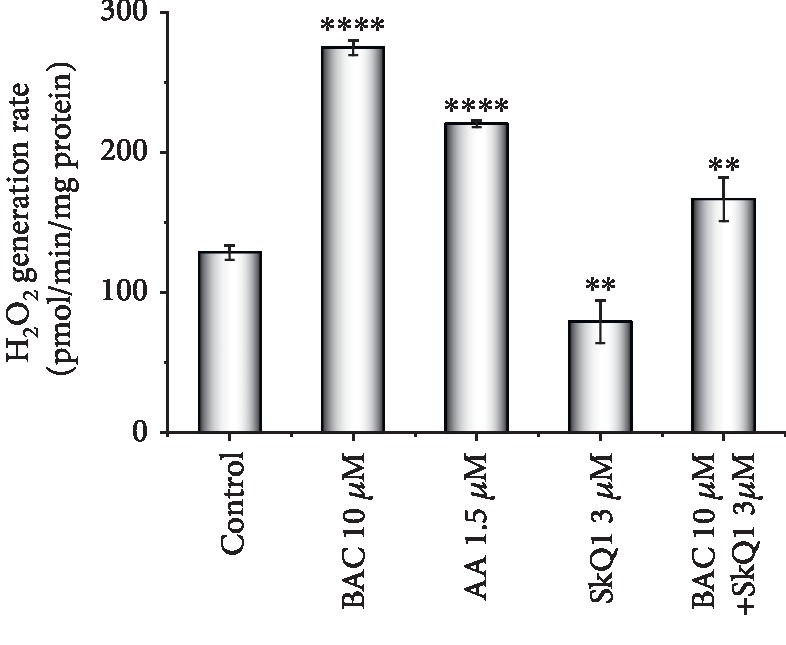
Hydrogen peroxide generation by rat liver mitochondria respiring on succinate. The basal incubation medium was supplemented with 0.5 mM EGTA, 20 mM Tris-succinate, 6 mM aminotriazole, 5 *μ*M Amplex Red, horseradish peroxidase (9 IU/ml), and mitochondria (0.25 mg protein/ml). The statistical analyses were carried out by the one-way ANOVA test against control. ^∗∗^0.001 < *p* < 0.01; ^∗∗∗∗^*p* < 0.0001.

Finally, we examined BAC effect on hydrogen peroxide production by rat liver mitochondria respiring on succinate (Figure 7). To prevent catalase activity, the medium was supplemented with 6 mM aminotriazole. Antimycin A, an inhibitor of electron transport in complex III of the respiratory chain, known to enhance hydrogen peroxide production by stimulating the reverse electron transfer, was taken as a positive control. Hydrogen peroxide production by mitochondria was almost doubled in the presence of 10 *μ*M BAC, attaining a level comparable to that exerted by 1.5 *μ*M antimycin A, clearly showing that BAC acted as a prooxidant. BAC-induced peroxide production by mitochondria was considerably diminished by SkQ1, a rechargeable mitochondria-targeted antioxidant [67].

### 3.8. BAC-Induced Oxidative Stress in the *Y. lipolytica* Yeast

**Figure 8:**
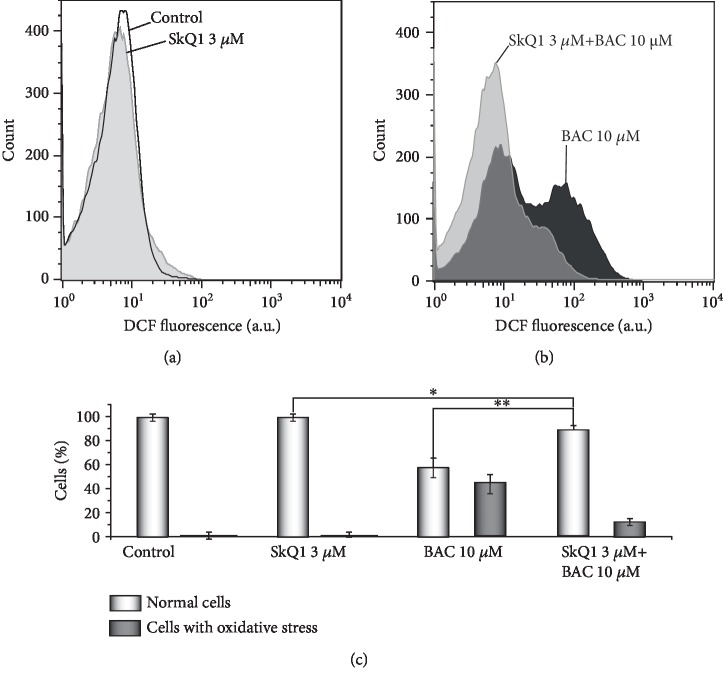
BAC-induced oxidative stress in the *Y*. *lipolytica* yeast. Exponentially grown yeast cells were either incubated with 10 *μ*M BAC for 2 h (b, marked by black) or initially preincubated with 3 *μ*M SkQ1 for 30 min and then incubated with 10 *μ*M BAC for 2 h (b, marked by light grey). All samples were rinsed with a fresh portion of growth medium and incubated with 15 *μ*M H_2_DCF-DA for 30 min in the dark. Stained cells were analyzed by flow cytometry. The statistical analyses were carried out by the one-way ANOVA test. ^∗∗^0.001 < *p* < 0.01; ^∗^0.01 < *p* < 0.05.

Production of intracellular ROS was detected with the use of DCF (for details, see Materials and Methods). In the control experiments (Figure 8(a)), SkQ1 only marginally decreased the DCF-related fluorescence of the control cells. Exposure of exponentially grown *Y. lipolytica* cells to 10 *μ*M BAC induced oxidative stress (Figure 8(b)), as inferred from a shift towards stronger DCF fluorescence (Figure 8(b), marked by black). The BAC-induced oxidative stress was largely prevented by SKQ1 (Figure 8(b), marked by light grey). In Figure 8(c), the data obtained are presented as diagrams.

### 3.9. BAC-Induced Fragmentation of Mitochondria in *D. magnusii* Cells

**Figure 9:**
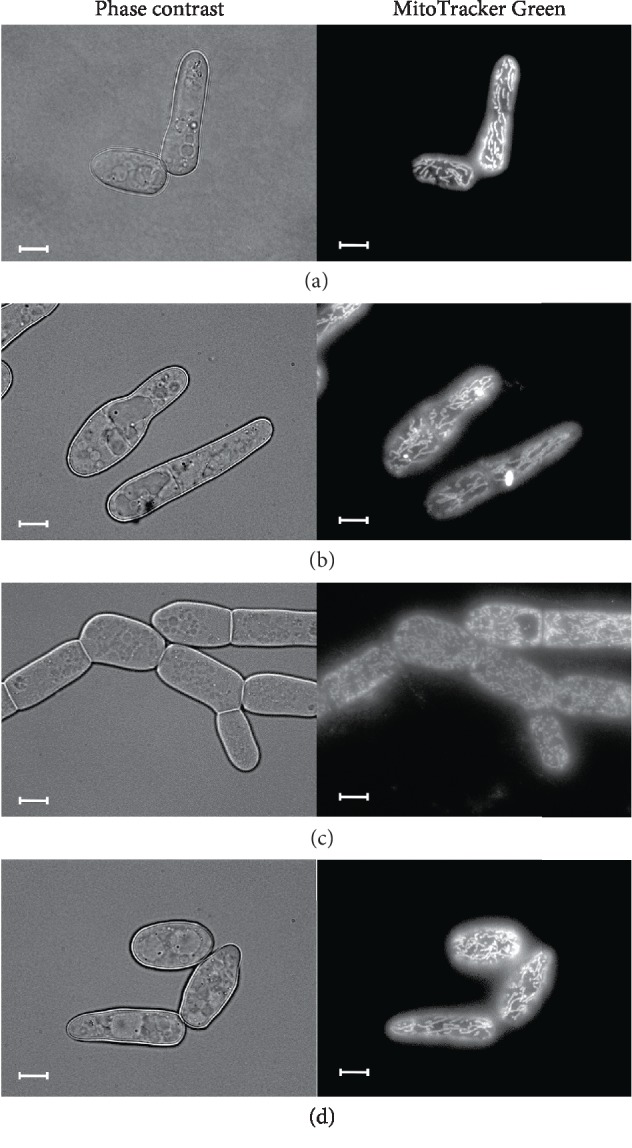
BAC-triggered mitochondria fragmentation in *D. magnusii* cells, the protective effect of SkQ1. Exponentially grown yeast cells were used. (a) Control cells; (b) cells were incubated with 800 nM SkQ1 for 1 h; (c) cells were incubated with 45 *μ*M BAC for 1 h; (d) cells were preincubated with 800 nM SkQ1 for 1 h, washed in 50 mM PBS buffer, pH 5.5, suspended in a fresh portion of growth medium and then incubated with 45 *μ*M BAC for 1 h. For visualization of mitochondria, cells were washed with 50 mM PBS, pH 5.5, and stained with 200 nM MitoTracker Green for 30 min. Scale bars are 10 *μ*m.

Normally, *D. magnusii* cells possess highly branched mitochondrial reticulum (Figure 9(a)). However, when cells were treated with 45 *μ*M BAC for 1 h, disruption of the integrity of this mitochondrial reticulum (mitochondrial fragmentation) took place (Figure 9(c)). A 1 h preincubation of yeast cells with SkQ1, living mitochondrial morphology unchanged (Figure 9(b)), partially prevented BAC-induced mitochondrial fragmentation (Figure 9(d)), presumably by alleviating the oxidative stress challenge.

**Figure 10:**
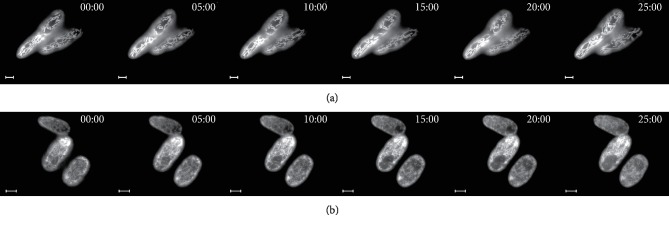
Time-lapse microscopy of *D. magnusii* cells. Exponentially grown yeast cells were used. For visualization of mitochondria, cells were rinsed with 50 mM PBS, pH 5.5, and then incubated with 200 nM MitoTracker Green for 30 min. (a) Control; cells were imaged for 25 min with a 30 s time interval. The full video is presented in Video 1 in the Supplementary Material. (b) Cells were incubated with 45 *μ*M BAC; cells were imaged for 25 min with a 30 s time interval. The full video is presented in Video 2 in the Supplementary Material. Scale bars are 10 *μ*m.

**Figure 11:**
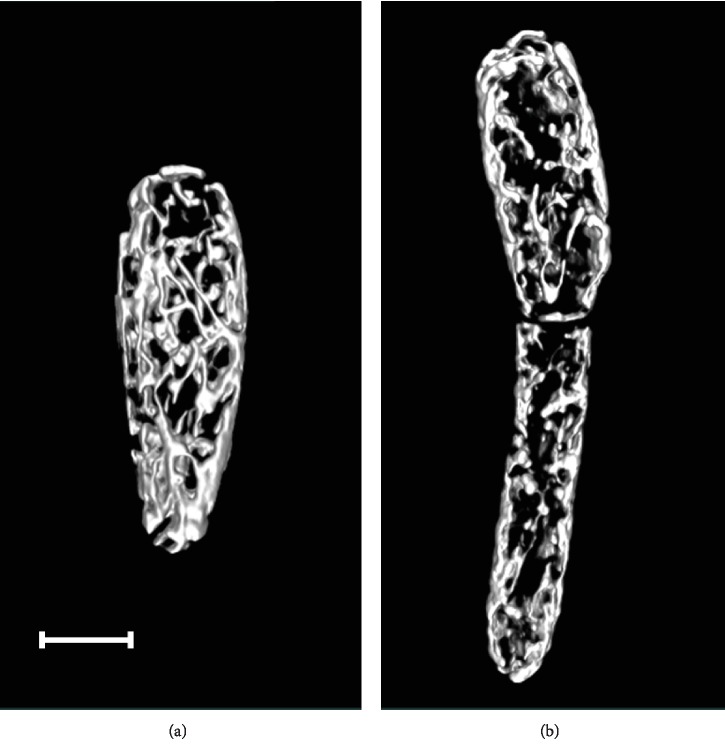
3D reconstruction of mitochondrial structures in *D*. *magnusii* cells. Exponentially grown yeast cells were incubated in (a) fresh portion of growth medium or (b) with additionally added 45 *μ*M BAC for 1 h. Then, cells were washed with 50 mM PBS, pH 5.5, and incubated with 200 nM MitoTracker Green for 30 min. The full video is presented in Video 3 in the Supplementary Material. Scale bars are 10 *μ*m.

To gain a better insight into mitochondrial structure and dynamics in real time in single *D. magnusii* cells, we have used time-lapse microscopy (Figure 10 and Videos [Supplementary-material supplementary-material-1] and [Supplementary-material supplementary-material-1] in the Supplementary Material) and 3D reconstruction of the yeast mitochondrial system (Figure 11 and [Supplementary-material supplementary-material-1] in the Supplementary Material). Both strategies reinforced the notions that under “normal” conditions *D. magnusii* cells retained mitochondrial reticulum (Figures 10(a) and 11(a), Videos [Supplementary-material supplementary-material-1] and [Supplementary-material supplementary-material-1]a in the Supplementary Material). The integrity of mitochondrial reticulum was lost when cells were exposed to 45 *μ*M BAC (Figures 10(b) and 11(b), Videos [Supplementary-material supplementary-material-1] and [Supplementary-material supplementary-material-1]b in the Supplementary Material).

## 4. Discussion

Over more than two decades, a large number of studies, using a variety of models, cells, and tissues, have demonstrated that BAC causes or enhances harmful consequences on the eye structures that may deeply impact patients' quality of life, compliance, or later surgical outcome (see Introduction). This situation is reinforced by the here-obtained results. Here, we showed that BAC, even at low concentrations, induced dysfunction of mitochondria, inhibiting respiration in state 3, decreasing the membrane potential, promoting opening of the mPTP, potentiating hydrogen peroxide production by mitochondria, and severely blocking synthesis of ATP. Previously, it was reported that BAC inhibited mitochondrial ATP production and oxygen consumption in human corneal epithelial cells and cells bearing LHON mutations possibly by directly targeting mitochondrial complex I [48]. However, closer examination of this paper showed that the authors used permeabilized cells as models and, more importantly, inadequate, from the point of view of bioenergetics, “mitochondrial buffer” causing high-amplitude swelling of mitochondria with all the consequences. This does not permit to consider these data as conclusive. In our approach, we applied as a model tightly coupled (high-quality) rat liver mitochondria that are a proper biosensor for assessment of drugs and specific directed agents. A comprehensive analysis of BAC effects on mitochondria showed that BAC did not exhibit an uncoupling activity (Figure 2), resembling in this respect the behaviour of other hydrophobic lipophilic quaternary ammonium compounds such as C_10_TEA and C_16_TMA investigated by us previously [66]. In the cited paper, these compounds, as exemplified by C_16_TMA, were found to possess high affinity to the membrane surface (the binding energy for C_16_TMA was equal to 48 kJ/mol), which apparently originated from its long hydrocarbon “tail,” and enhanced activation energy (for C_16_TMA, 71 kJ/mol), and hence lowered permeability through lipid bilayer, possibly due to the small size of their charged “head.” Importantly, C_10_TEA and C_16_TMA were inactive in the BLM test (no diffusion electric potential was generated). Assuming that C_10_TEA, C_16_TMA, and BAC share similar properties (they are hydrophobic lipophilic quaternary ammonium compounds with screening of positive charge of lipophilic cations by bulky residues), this might explain the lack of uncoupling activity exerted by BAC (Figure 2), as well as the development of its inhibitory effect with time (Figures 2 and 3). Most probably, depolarizing activity (Δ*ψ* decrease) of BAC was due to its inhibitory effect on the respiratory chain (Figure 4).

The most pronounced inhibitory effect of BAC on mitochondria was revealed when testing ATP synthesis (Figure 6). It should be noted that the rate of ATP synthesis is an integral parameter, dependent not only on ATP synthase activity itself but also on activity of the ADP/ATP translocase, importing ADP into the mitochondrion for ATP synthesis and exporting the synthesized ATP out of the mitochondrion for use in the cytosol. ADP/ATP translocase is the most abundant protein of the mitochondrial inner membrane, with two active centers exposed on both sites of the inner mitochondrial membrane, including the outer one [68]. The precise mechanism of inhibitory action of BAC on the translocase is unknown; this issue in the literature has been totally ignored. Keeping in mind, by analogy to C_16_TMA, a lowered permeability of BAC through lipid bilayer, we would like to speculate that the mitochondrial ADP/ATP in the state “c conformation” (with the active center opened on the cytoplasmic side of the inner membrane) might be one of the major targets of BAC in mitochondria, most accessible for direct interaction with BAC. We are inclined to think that BAC inhibitory effect on the ADP/ATP translocase might be similar to its antimicrobial action, being associated with insertion of BAC alkyl group into the hydrophobic core of the inner mitochondrial membrane, thus disturbing the membrane and the translocase dominating in the membrane. As the ADP/ATP translocase, in addition to its normal function, forms the inner membrane channel of the mitochondrial permeability transition pore [68], a disturbance of the translocase would affect also the pore (Figure 5). BAC was found to trigger oxidative stress in mitochondria that was largely mitigated by the antioxidant SkQ1 (Figure 7). Undoubtedly, further experiments are needed to gain a better understanding of the inhibitory effects of BAC on mitochondria.

Other models utilized in this study were yeast cells with a mode of energy conservation closely similar to that of higher organisms. Yeast cells are appropriate (adequate) models for such type of investigations as they possess most of the same fundamental cellular signaling pathways that regulate metabolism, cell growth and division, organelle function, cellular homeostasis, and stress responses. Moreover, thanks to the amenability of yeast cells to both classical and advanced molecular genetic techniques, to relatively simple, cheap, and quick genetic and environmental manipulations, to the large knowledge base and data collections, high-throughput screening technologies, and functional genomics that are not possible in humans, yeasts have become valuable and prevalent eukaryotic model organisms to unravel complex fundamental intracellular mechanisms underlying human homeostasis and pathologies. BAC in yeast cells exerted pronounced prooxidant effect (Figure 8) and triggered mitochondrial fragmentation (Figures 9–11). The latter observation deserves a special attention. Mitochondria in various eukaryotes are undergoing well-orchestrated fission and fusion [52], and this organellar dynamics, as a part of the quality control mechanism, has the power to restore the function of impaired organelles by content mixing with intact organelles. Moreover, the excessive mitochondrial fission (fragmentation), like a mitochondrial bioenergy deficit, has been documented as the earliest feature preceding appearance of known hallmarks of pathologies, in Alzheimer disease, for example [69].

Damaged action of BAC on rat liver mitochondria and yeast cells associated with its prooxidant effect can be considerably diminished by SkQ1, a mitochondria-addressed (transported exclusively in mitochondria) antioxidant (Figures 7 and 8). These promising results may be considered as a rationale for advisability of using mitochondrial-targeted antioxidants in ophthalmic formulations containing BAC and, more generally, for mitigating diseases related to oxidative stress and mitochondrial dysfunctions [70–74].

Many *in vivo* and *in vitro* studies have been developed to alleviate [29, 36, 75–80], to predict or even reverse [21] the toxic effects of BAC, to replace it for other less toxic preservatives [7, 26, 32, 81–89], or to remove BAC from formulations before the use [90].

Several findings indicate that extensive use of BAC as a disinfectant could lead to emergence of antibiotic-resistant isolates through the induction of cross-resistance, which may have important implications for both human and environmental health [21, 91–94].

## 5. Conclusion

We believe that now there are more sound reasons to suggest that mitochondrial dysfunction, including lowered respiration, decreased membrane potential, opening of the mPTP, and diminished ATP production, in concert with oxidative stress and disruption of mitochondrial reticulum (mitochondrial fragmentation) may culminate BAC-induced harmful effects than this could have been assumed before [32].

## Data Availability

The data used to support the findings of this study are available from the corresponding author upon request.

## Abbreviations

Δ*ψ*: Transmembrane electric potential difference
Ap5A: P1,P5-di(adenosine-5′) pentaphosphate
BAC: Benzalkonium chloride
BLM: Bilayer planar phospholipid membrane
BSA: Bovine serum albumin
C_10_TEA: Decyltriethylammonium bromide
C_16_TMA: Cetyltrimethylammonium bromide
CCCP: Carbonylcyanide m-chlorophenylhydrazone
CsA: Cyclosporine A
DCF-DA: 2′,7′-Dichlorodihydrofluorescein diacetate
EGTA: Ethylene glycol-bis(aminoethyl ether) N,N,N′,N′-tetraacetic acid
mPTP: Ca^2+^/Pi-dependent mitochondrial permeability transition pore
Oligo: Oligomycin
PI: Propidium iodide
ROS: Reactive oxygen species
SkQ1: 10-(6′-Plastoquinonyl)decyltriphenylphosphonium.

## Supplementary Materials

Supplementary 1 Video 1. Time-lapse microscopy of *D. magnusii* control cells. Yeast cells were grown as described in Materials and Methods. For visualization of mitochondria, cells were rinsed by 50 mM PBS, pH 5.5, and then incubated with 200 nM MitoTracker Green for 30 min. Cells were imaged for 25 min with a 30 s time interval. Scale bars are 10 *μ*m.

Supplementary 2 Video 2. Time-lapse microscopy of *D. magnusii* cells incubated with 45 *μ*M BAC. Yeast cells were grown as described in Materials and Methods. For visualization of mitochondria, cells were rinsed by 50 mM PBS, pH 5.5, and then incubated with 200 nM MitoTracker Green for 30 min. Cells were imaged for 25 min with a 30 s time interval. Scale bars are 10 *μ*m.

Supplementary 3 Video 3. 3D reconstruction of mitochondrial structures in *D. magnusii* cells. Exponentially grown yeast cells were incubated in a fresh portion of growth medium (a) or with additionally added 45 *μ*M BAC (b) for 1 h. Then, cells were washed by 50 mM PBS, pH 5.5, and incubated with 200 nM MitoTracker Green for 30 min. Scale bars are 10 *μ*m.
